# VPAC receptor positivity in comparison with mp‐MRI in the diagnosis of prostate cancer: A preliminary study

**DOI:** 10.1002/bco2.70006

**Published:** 2025-04-22

**Authors:** Nishant Setya, Shridhar C. Ghagane, Rajendra B. Nerli, Ashwin Bokare, Madhukar L. Thakur, Leonard Gomella

**Affiliations:** ^1^ Department of Urology, J.N. Medical College KLE Academy of Higher Education and Research (Deemed‐to‐be‐University) Belagavi India; ^2^ KAHER's Dr. Prabhakar Kore Basic Science Research Center KLE Academy of Higher Education and Research (Deemed‐to‐be‐University) Belagavi India; ^3^ Division of Urologic‐oncology, Urinary Biomarkers Research Centre KLES Dr. Prabhakar Kore Hospital and Medical Research Centre Belagavi India; ^4^ Departments of Urology, Radiology Thomas Jefferson University Philadelphia Pennsylvania USA; ^5^ Departments of Urology Thomas Jefferson University Philadelphia Pennsylvania USA; ^6^ Sidney Kimmel Cancer Centre Thomas Jefferson University Philadelphia Pennsylvania USA

**Keywords:** imaging, optical imaging, prostate cancer, urinary assay, VPAC receptors

## Abstract

**Objective:**

The study aimed to prospectively evaluate the feasibility of diagnosing PCa using voided urine samples and by targeting the genomic VPAC (vasoactive intestinal peptide and pituitary adenylate cyclase‐activating peptide) receptors in comparison with multiparametric magnetic resonance imaging (mp‐MRI) in male patients (≥40 years of age) with lower urinary tract symptoms and having a serum PSA of >4 but <15 ng/ml.

**Patients and Methods:**

Male patients attending urological services ≥40 years old, with lower urinary tract symptoms and serum PSA levels of >4 but <15 ng/ml formed the study group. Voided urine samples were collected to target VPAC receptors on malignant cells. All patients underwent mp‐MRI. A 12‐core transrectal ultrasound‐guided prostate biopsy was performed in all, and the results were compared for the diagnosis of PCa.

**Results:**

A total of 61 patients with a median age of 65.33 ± 8.11 years and with a median serum PSA of 9.56 ± 2.78 ng/ml were further evaluated with both urinary biomarker assessment and mp‐MRI. Histopathological (HPR) confirmation of PCa was noted in 25 (40.98%) patients and benign prostatic hyperplasia in the remaining 36 (59.01%) patients. Of the 25 patients with histologically proven PCa, the urinary biomarker (VPAC positivity) was positive for malignancy in 24 (96%), one case showed false negative results (4%) and there were no false positive cases (0%). HPR confirming PCa was seen in 3/16 patients with a PIRADS 2 score, 7/21 patients with a PIRADS 3 score, 7/14 patients with a PIRADS 4 score and 8/8 patients with a PIRADS score of 5.

**Conclusions:**

VPAC receptor positivity of prostate cancer cells is an easy test to perform using a voided urine sample. VPAC receptor positivity can be used as an indication for prostate biopsy in patients having a negative previous biopsy but highly suspicious of cancer, in patients with an elevated serum PSA but with a normal digital rectal examination and in patients with benign features and borderline elevation of serum PSA.

## 1 INTRODUCTION

Diagnosis of prostate cancer (PCa) plays a fundamental role in ensuring that an appropriate therapeutic option is offered, whilst, at the same time, ensuring prevention of overdiagnosis and overtreatment.^1^ An elevated PSA alone should no longer be an indication for a prostate biopsy. The use of diagnostic adjuncts can help to predict the presence of clinically significant prostate cancer thereby avoiding unnecessary biopsies in a proportion of patients.^2^

Digital Rectal Examination can be used as an inexpensive diagnostic tool to check the prostate for cancer and to give an assessment of the prostate volume. However, there is a high degree of interobserver variability, and a normal DRE does not eliminate the risk of significant prostate cancer.^3^ Higher levels of PSA indicate a greater likelihood of prostate cancer. A PSA cut‐off of ≤4 ng/ml was originally proposed as a normal level in men aged 50–70 years.^4^ The Prostate Cancer Prevention Trial (PCPT) however found that 15% of men with a PSA level of ≤4.0 ng/ml had clinically significant prostate cancer.^5^ A population‐based study found that 30% of men with an abnormal PSA had a return to normal PSA on their next reading.^6^

An increased PSA density was associated with a higher risk of prostate cancer, with a generally agreed cut‐off value of between 0.12 and 0.15 ng/ml/cc.^7^, ^8^ The prostate health index has been shown to have greater specificity and sensitivity than any of its components.^9^, ^10^ The use of Phi has the potential to reduce unnecessary biopsies. The 4K score has also been shown to be a predictor for prostate cancer, which can be used to avoid unnecessary biopsies.^11^, ^12^ A direct comparison of the 4K score and Phi found both tests to be equally predictive of prostate cancer and clinically significant prostate cancer.^13^

mp‐MRI should include a combination of high‐resolution T2‐weighted images and at least two functional MRI techniques: diffusion‐weighted imaging (DWI) and dynamic contrast‐enhanced (DCE) imaging.^14^ Prostate cancer typically manifests as a round low signal intensity focus on T2‐weighted MRI, high signal intensity on DWI at high *b* values and classically demonstrates early enhancement on DCE‐MRI. The Prostate Imaging‐Reporting and Data System (PI‐RADS) provides a structured way to report each lesion by allocating a score between 1 and 5 that predicts its chance of being a clinically significant prostate cancer, with 5 indicating a very high likelihood for the presence of clinically significant prostate cancer.^15^ A meta‐analysis assessing the diagnostic accuracy of mp‐MRI for prostate cancer found it to have high specificity and sensitivity, 88% and 74%, with a variable but high negative predictive value ranging from 65% to 94%.^16^

Several urinary biomarkers for prostate cancer have also been described, which include urinary measurements of prostate cancer gene 3, Transmembrane protease serine 2 genes (*TMPRSS2: ERG*) and SelectMDX test.^1^ Cristofanilli et al.^17^ used voided urine samples to non‐invasively diagnose prostate cancer using a simple and reliable assay and by targeting genomic vasoactive intestinal peptide and pituitary adenylate cyclase‐activating peptide (VPAC) receptors expressed on malignant PCa cells shed in voided urine. Similarly, Reubi et al.^18^ assessed the feasibility of detecting PCa using voided urine by targeting the genomic VPAC receptors expressed on malignant PCa cells in 75 patients. In this prospective study, we have assessed the feasibility of diagnosing PCa using voided urine samples and by targeting the genomic VPAC receptors expressed on the malignant cancer cells in comparison with mp‐MRI in male patients (≥40 years of age) with lower urinary tract symptoms and having a serum PSA of >4 but <15 ng/ml.

## 2 PATIENTS AND METHODS

This prospective study was undertaken following consent obtained from the University/Institutional ethical committee Ref No. MDC/JNMCIEC/165 dated: 17/03/2023. Male patients attending urological services ≥40 years old, with lower urinary tract symptoms and serum PSA levels of >4 but <15 ng/ml, formed the study group. Patients with urinary tract infection, hematuria, history of urothelial carcinoma, and radiotherapy were excluded from the study. The first 50 ml of voided urine sample was collected from all the patients in the study group, to be used for biomarker studies. The urine samples were stored and processed at 22°C for up to 4 h. However, if the processing was likely to be delayed due to some reason for more than 4 h of urine collection, then the samples were stored at −20°C for up to 72 h.

### 2.1 Processing of urine samples

These samples were labelled with the hospital identification number, date of collection and clinical diagnosis. The samples of urine were centrifuged using a cytocentrifuge 2000×*g* for 10 min. Except for 250 μl of supernatant, the rest was discarded. The cells were then resuspended, cytocentrifuged and fixed in 97% ethanol. A cell area of approximately 1 cm in diameter was covered with TP4303 solution (0.5 μg). The slide was then incubated in the dark, at 22°C for 20 min, and then thoroughly rinsed with deionized water, and the slide was air dried. The dried slide was then added with 20 μl of 4,6‐dimidino‐2‐phenylindole, dihydrochloride (DAPI) (Fisher Scientific, PA), which strongly binds to A‐T rich region of DNA in the cell nucleus.^17^, ^18^

The slide was covered with a coverslip and observed using a fluorescent microscope. Cells stained with TP4303 interaction appeared with dark orange fluorescence around the nucleus and thereby indicated the presence of VPAC molecules around the cell surface. In the absence of VPAC, the cell nucleus that was bound only with DAPI appeared dark blue. However, normal epithelial cells that may only have minimal or no expression of VPAC receptors do not interact with TP4303 and therefore showed only cell nuclei. Urine specimens showing positivity of VPAC receptors were considered positive for malignancy.

The patients in the study group underwent mp‐MRI imaging of the prostate and bony pelvis. The images were read and reported by the same group of radiologists. PI‐RADS scoring system was used to classify the results of mp‐MRI. Patients having a score of 3–5 were considered suspicious of PCa. All patients in the study group further underwent a standard 12‐core prostate biopsy using transrectal ultrasonography (TRUS) guidance, and all those patients with high suspicion on mpMRI (PIRADS 3–5) also underwent targeted biopsies from those areas. The specimens were labelled properly denoting the zone of the prostate and sent for histopathological estimation. The results of the histopathological studies were then compared to the results of urine biomarker studies and the results of mp‐MRI imaging.

## 3 RESULTS

During the study period, a total of 61 patients with a median age of 65.33 ± 8.11 years and with a median serum PSA of 9.56 ± 2.78 ng/ml were further evaluated with both urinary biomarker assessment as well as mp‐MRI. DRE of the prostate revealed abnormal findings in only five (8.19%) patients, which included hard consistency of one of the lobes of the prostate in three patients and a nodule in two other patients. Of the 61 patients, a histopathological confirmation of PCa was seen in 25 patients. The rest 39 patients showed no evidence of cancer, and all confirmed the presence of Benign prostatic hyperplasia (BPH). All the patients with HPR‐proven PCa had significant cancer (≥7 Gleason's score and Gleason group grade ≥2).

**FIGURE 1:**
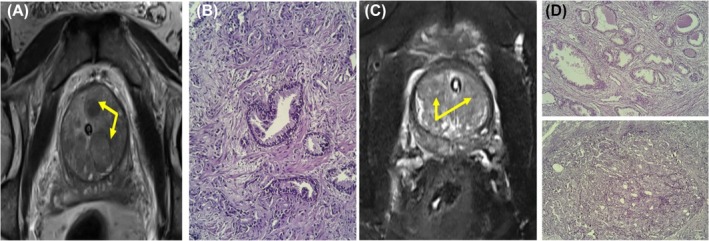
A 71‐year‐old male with a PSA of 8 ng/ml. (1A) Multiple T2 hypointense nodules in the transition zone well encapsulated with a thinned‐out peripheral zone suggestive of PIRADS‐2. (1B) Widely spaced small‐sized irregularly shaped neoplastic glands (Gleason Pattern 3) with focal areas of cellular sheeting (Gleason Pattern 4) within a desmoplastic stroma. The cells show moderate nuclear atypia and hyperchromatic nuclei suggestive of Gleason Grade Group 3. A 67‐year‐old male with PSA of 10.2 ng/ml. (1C) Capsulated mildly T2 hypointense nodules in the transition zone suggestive of PIRADS‐2. (1d) Widely spaced sized irregularly shaped neoplastic glands (Gleason Pattern 3) with focal areas of cellular sheeting (Gleason Pattern 4) within a desmoplastic stroma. The cells show moderate nuclear atypia and hyperchromatic nuclei suggestive of Gleason Grade Group 3.

**FIGURE 2:**
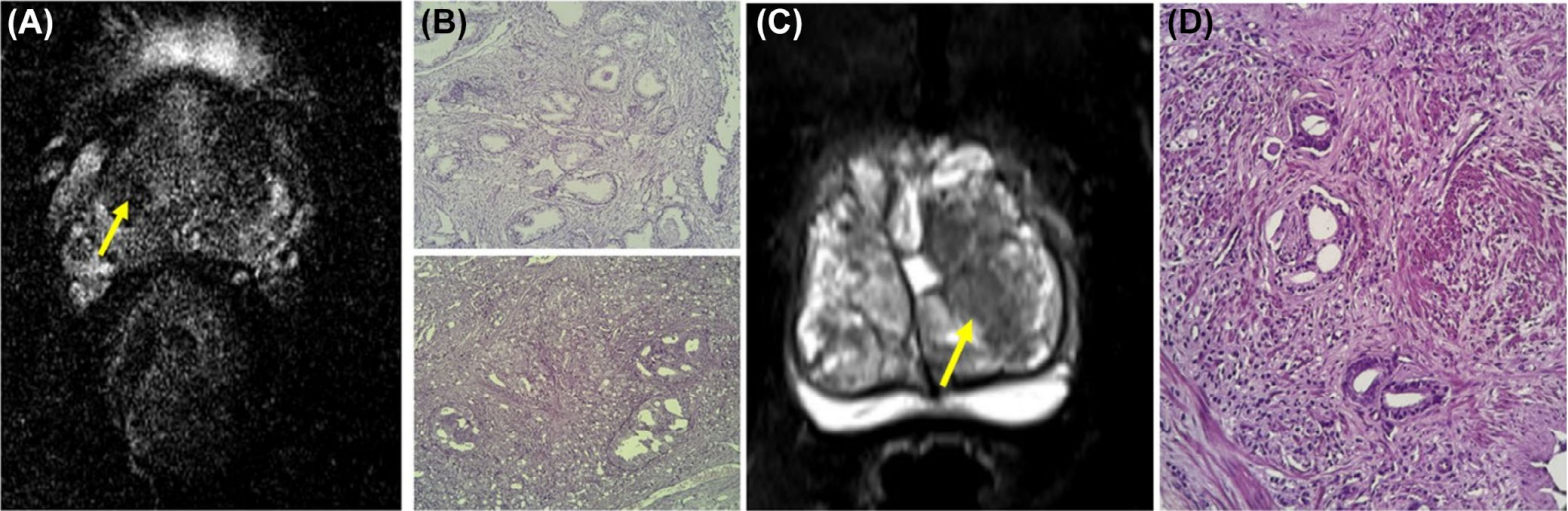
A 78‐year‐old male with 15 ng/ml PSA. (2A) Wedge shaped T2 hypointensity noted in transition zone suggestive of PIRADS‐3. (2B) Widely spaced sized irregularly shaped neoplastic glands (Gleason Pattern 3) with focal areas of cellular sheeting (Gleason Pattern 4) within a desmoplastic stroma. The cells show moderate nuclear atypia and hyperchromatic nuclei suggestive of Gleason Grade Group 3. A 65‐year‐old male with 14.1 ng/ml PSA. (2C) A T2 hypointense area of size 1.4 cm in the transitional zone with indistinct margins size suggestive of PIRADS‐4. (2D) Infiltrative sheets (Gleason Pattern 5) of neoplastic cells with occasional glandular formations (Gleason Pattern 4). The stroma is desmoplastic with splaying of muscle fibres. The cells show moderate nuclear atypia and hyperchromatic nuclei. Foci of perineurial invasion are also seen as suggestive of Gleason Grade Group 5.

**FIGURE 3:**
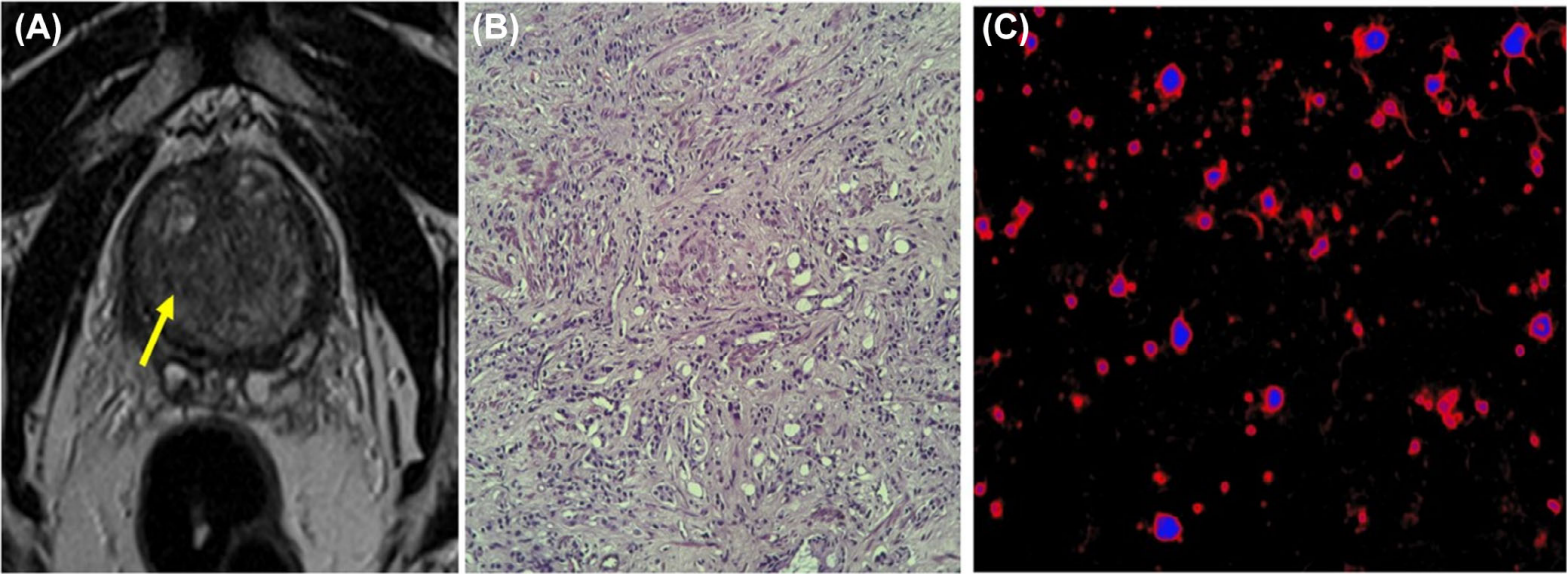
A 53‐year‐old male with 14 ng/ml PSA. (3A) A 1.5 cm focus of low T2 signal posterolaterally in the right peripheral zone at the level of the mid gland. With ADC restriction suggestive of PIRADS‐5. (3b) Infiltrative sheets of neoplastic cells in streams and sheets (Gleason Pattern 5) within a desmoplastic stroma and no glandular formation. There is moderate nuclear atypia and hyperchromatic nuclei suggestive of Gleason Grade Group 5. (3c) Cells positive for VPAC receptor suggestive of malignancy.

**FIGURE 4:**
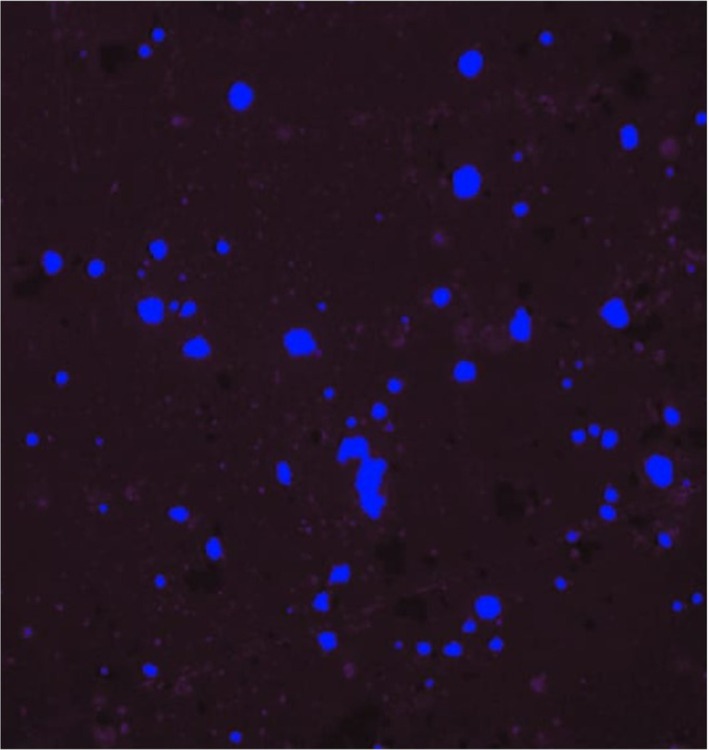
Normal epithelial cells with blue nuclei after staining that had minimal or no expression of VPAC receptors.

Of the 25 patients with histologically proven PCa, the urinary biomarker (VPAC positivity) was positive for malignancy in 24 (96%), one case showed false negative results (4%), and there were no false positive cases (0%). mp‐MRI findings are mentioned in (Tables 1 and 2). The relationship between Gleason's group grade and PIRADS results is shown in Table 3. There was a linear relationship observed between Serum PSA and Gleason's group grade. Comparison between Urinary Biomarker Test and TRUS Biopsy results is shown in Table 4. The measures of diagnostic accuracy of mpMRI shows the sensitivity: 0.88 (CI: 0.753–1); specificity: 0.42 (CI: 0.256–0.578); PPV: 0.51 (CI: 0.362–0.661); and NPV: 0.83 (CI: 0.661–1). The measures of diagnostic accuracy of UBT show sensitivity: 0.96 (CI: 0.883–1); specificity: 1 (CI: 1–1); PPV: 1 (CI: 1–1); and NPV: 0.97 (CI: 0.921–1). The same has been depicted in Table 5. The comparison of histopathological studies, mp‐MRI imaging and results of urine biomarkers of selected patients are shown in (Figures 1, 2, 3). The result of the negative staining report is shown in Figure 4.

**TABLE 1:** mp‐MRI and histopathological findings.

PIRADS score	No. of patients	Histopathological positive (%)	Histopathological negative (%)
1	2 (3.27)	–	2 (5.55)
2	16 (26.22)	3 (12)	13 (36.11)
3	21 (34.42)	7 (28)	14 (38.8)
4	14 (22.95)	7 (28)	7 (19.4)
5	8 (13.11)	8 (32)	–
Total	61	25	36

**TABLE 2:** Comparison between PIRADS score (mp‐MRI) and TRUS biopsy.

mp‐MRI	TRUS guided prostate biopsy	
	Positive (%)	Negative (%)
PIRADS 3/4/5 (43)	22 (36.06)	21 (34.42)
PIRADS 1/2 (18)	3 (4.91)	15 (24.59)
Total (61)	25	36

**TABLE 3:** Gleason's group grade and PIRADS.

Gleason group gr	PIRADS 1	PIRADS 2	PIRADS 3	PIRADS 4	PIRADS 5
2	–	1	1	1	–
3	–	2	1	3	1
4	–	–	3	2	3
5	–	–	2	1	4
Total	0	3 (12%)	7 (28%)	7 (28%)	8 (32%)

**TABLE 4:** Comparison between urinary biomarker test and TRUS biopsy results.

UBT	TRUS guided prostate biopsy	
	Positive (%)	Negative (%)
Positive (24)	24 (39.34)	0
Negative (37)	1 (1.63)	36 (59.01)
Total (61)	25	36

**TABLE 5:** Measures of diagnostic accuracy.

	mp‐MRI (%)	UBT (%)
Sensitivity	88	96
Positive predictive value	51	100
Specificity	42	100
Negative predictive value	83	97

## 4 DISCUSSION

Prostate cancer is the second most commonly diagnosed cancer and the fifth leading cause of cancer death among men worldwide, with an estimated 1 414 000 new cancer cases and 375 304 deaths in 2020.^19^ It is also worth noting at the same time that the burden of prostate cancer is on the rise, owing to the ageing population and economic growth,^20^ even in India.^21^ The development of, several novel, life‐sciences technologies has been paved by the better understanding of human diseases at cellular and molecular levels. Detection of circulating tumour cells (CTC) in human blood has drawn considerable attention and is rapidly advancing into clinical applications in the form of liquid biopsy,^22^, ^23^ which is based upon a collection of body fluids and cell isolation, followed by multiplex genomic profiling and identification of disease‐specific fingerprints.

The human VPAC1 receptor, named for the combined vasoactive intestinal peptide (VIP) and pituitary adenylate cyclase‐activating peptide (PACAP) family of cell surface receptors, encodes a G protein‐coupled receptor that recognizes with high affinity both VIP and PACAP related peptides. It has been demonstrated that VPAC1 receptors are expressed in men with prostate cancer irrespective of their hormonal status.^24^ High expression of VPAC1 receptors (10^4^/cell) in prostate cancer has been confirmed by Nerli et al.^25^, ^26^ Fütterer et al.^27^ imaged prostate cancers in humans with >97% sensitivity, using a Cu‐64 labelled VPAC1 specific peptide designed in their laboratories. Cristofanilli et al.^17^ hypothesized that cells shed in voided urine of PCa patients could be imaged optically, by targeting the VPAC1 receptors with the same peptide labelled with a fluorophore. Cristofanilli et al.^17^ reported on their pilot study wherein an assay detected VPAC‐positive cells in 98.6% of the patients having a PCa diagnosis (*n* = 141) and none (0%) of the males with benign prostatic hyperplasia (BPH) (*n* = 10). The sensitivity of the assay was 99.3% with a confidence interval of 96.1–100%, and the specificity was 100% with a confidence interval of 69.2–100%.

The VPAC genomic biomarker belongs to the superfamily of G‐protein coupled surface receptors, which are expressed in high density (10^4^–10^5^/cell) at the onset of oncogenesis and before the alterations in cell morphology.^28^, ^29^ VPAC1 receptors are minimally present on the stroma, normal cells and benign masses.^26^, ^30^ VPAC receptors are also expressed in breast and lung cancer.^28^ Nerli et al. have reported that VPAC receptors are also expressed on malignant cells from carcinoma of the cervix,^24^ transitional cell carcinoma of the bladder and squamous cell carcinoma of the oral cavity.^25^

Multiparametric prostate MRI offers a non‐invasive approach to detect prostate cancer. In 2012, the European Society of Urogenital Radiology developed the first standardized guidelines for prostate MRI, aiming to enhance the accuracy of radiologic interpretations.^15^ In 2019, further refinements and assessments were carried out that resulted in the latest version, PIRADSv2.1.^15^ T2‐weighted images in combination with two functional sequences have been shown to provide better characterization of tumour's in the prostate. Hessels and Schalken^16^ in a diagnostic meta‐analysis of seven studies revealed high overall sensitivity and specificity on accuracy of mp‐MRI using T2WI, DWI and DCE MRI. Pooled sensitivity and specificity were 0.74 and 0.88, respectively, with negative predictive value (NPV) ranging from 0.65 to 0.94. Abd‐Alazeez et al.^30^ in another study showed that mp‐MRI was good at detecting and ruling out clinically significant cancer, following at least one previous biopsy, with an NPV of 95% using trans‐perineal template systemic biopsy as the gold standard.

Our study has clearly shown that targeting VPAC receptors on malignant cells that are present in the voided urine sample is far superior to performing a mp‐MRI for making a clinical diagnosis of significant PCa in men with a serum PSA of >4 but <15 ng/ml. This would help in selecting patients with an elevated serum PSA but having a normal DRE for a prostatic biopsy. Similarly, it would help in indicating a biopsy in patients with a marginally elevated PSA or patients with a clinical diagnosis of BPH. This test would also help in those cases where a biopsy is negative for cancer, but there is a strong indication for a repeat biopsy. We all know that a prostate biopsy be it transrectal or transperineal is associated with several complications that include, per‐rectal bleeds, pain, vasovagal symptoms, hematospermia, fever, dysuria, macroscopic hematuria, urinary tract infection and even septicaemia, which can be associated with morbidity and even deaths. Performing a test on the voided urine sample is free of these complications, is cheap, avoids a need for hospital admission in some cases, would be cheaper and could be repeated many times.

Our study clearly shows that if the VPAC receptor positivity was an indication for prostate biopsy then, an unnecessary biopsy would be performed in none of the patients with a benign condition. With the positive predictive value being 96%, only 4% of patients would have had a false negative prediction. However, in cases of mp‐MRI, as seen in our study, an unnecessary biopsy would have been performed in 21/43 (48.83%) if PIRADS 3–5 were taken as an indication for a biopsy, and an unnecessary biopsy would have been performed in 7/15 (46.66%) patients if only PIRADS 4 and 5 were taken as an indication for a prostate biopsy. Moreover, cancers would have been missed in 3/16 (18.75%) patients with PIRADS 2 reports wherein a biopsy would not have been indicated. PIRADS reporting though standardized can still be subjective, whereas the reporting of VPAC receptor positivity is standardized. Malignant cells show an orange cell wall surrounding a blue nucleus on fluorescent microscopy each colour representing a different wavelength. The shortcoming of our study is that this study is a single‐centre study and TP 4303 the flurophobe is still a research molecule awaiting commercialization. Multicentric studies would be able to confirm the success of targeting the VPAC receptor for making a diagnosis of PCa.

## 5 CONCLUSION

VPAC receptor positivity of prostate cancer cells is an easy test to perform using a voided urine sample. VPAC receptor positivity can be used as an indication for prostate biopsy in patients having a negative previous biopsy but highly suspicious of cancer, in patients with an elevated serum PSA but with a normal digital rectal examination and in patients with benign features and borderline elevation of serum PSA.

## AUTHOR CONTRIBUTIONS

NS and SCG performed the laboratory, clinical experiments and statistical analysis. RBN & SCG conceptualized the study design and drafted the manuscript. AB collected the clinical data. MLT and LG supported the patented molecule for clinical validation and supervised the study. All the authors reviewed, edited and approved the manuscript.

## ACKNOWLEDGEMENTS

The authors are grateful to the patients for providing the clinical samples for the study. The authors are also thankful to the authorities of the organization for providing the infrastructure to conduct this study.

## CONFLICT OF INTEREST STATEMENT

Dr. Madhukar L. Thakur and Dr. Leonard Gomella hold patents with Thomas Jefferson University for the product/TP4303 molecule reported in this study.

## References

[1] Sharma M , Nerli RB , Nutalapati SH , Ghagane SC . Hypoehoic lesions on Transrectal ultrasound and its correlation to Gleason grade in the diagnosis of clinically significant prostate cancer: a prospective study. S Asian J Cancer. 2021;10(3):155–160. 10.1055/s-0041-1731906 PMC8687870 34938677

[2] Thompson IM , Pauller DK , Goodman PJ , Tangen CM , Scott Lucia M , Parnes HL , et al. Prevalence of prostate cancer among men with a prostate‐specific antigen level < or = 4.0ng per millilitre. N Engl J Med. 2004;350(22):2239–2246. 10.1056/NEJMoa031918 15163773

[3] Ghagane SC , Nerli RB , Hiremath MB , Wagh AT , Magdum PV . Incidence of prostate cancer at a single tertiary care center in north Karnataka. Indian J Cancer. 2016;53(3):429–431. 10.4103/0019-509X.200671 28244476

[4] Jue JS , Barboza MP , Prakash NS , Venkatramani V , Sinha VR , Pavan N , et al. Re‐examining prostate‐specific antigen (PSA) density: defining the optimal PSA range and patients for using PSA density to predict prostate cancer using extended template biopsy. Oncology. 2017;105:123–128. 10.1016/j.urology.2017.04.015 28431993

[5] Catalona WJ , Partin AW , Sanda MG , Wei JT , Klee GG , Bangma CH , et al. A multicentre study of [−2] pro‐prostate specific antigen combined with prostate‐specific antigen and free prostate‐specific antigen for prostate cancer detection in the 2.0 to 10.0ng/ml prostate specific antigen range. J Urol. 2011;185(5):1650–1655. 10.1016/j.juro.2010.12.032 21419439 PMC3140702

[6] Loeb S , Sanda MG , Broyles DL , Shin SS , Bangma CH , Wei JT , et al. The prostate health index selectively identifies clinically significant prostate cancer. J Urol. 2015;193(4):1163–1169. 10.1016/j.juro.2014.10.121 25463993 PMC4404198

[7] Parekh DJ , Punnen S , Sjoberg DD , Asroff SW , Bailen JL , Cochran JS , et al. A multi‐institutional prospective trial in the USA confirms that the 4Kscore accurately identifies men with high‐grade prostate cancer. Eur Urol. 2015;68(3):464–470. 10.1016/j.eururo.2014.10.021 25454615

[8] Nordström T , Vickers A , Assel M , Lilja H , Grönberg H , Eklund M . Comparison between the four‐kallikrein panel and prostate health index for predicting prostate cancer. Eur Urol. 2015;68(1):139–146. 10.1016/j.eururo.2014.08.010 25151013 PMC4503229

[9] Barentsz JO , Richenberg J , Clements R , Choyke P , Verma S , Villeirs G , et al. ESUR prostate MR guidelines 2012. Eur Radiol. 2012;22(4):746–757. 10.1007/s00330-011-2377-y 22322308 PMC3297750

[10] Davenport MS , Downs E , George AK , Curci NE , Salka BR , Sullivan TQ , et al. Prostate imaging and data reporting system version 2 as a radiology performance metric: an analysis of 18 abdominal radiologists. J am Coll Radiol. 2021;18(8):1069–1076. 10.1016/j.jacr.2021.02.032 33848507

[11] de Rooij M , Hamoen EHJ , Fütterer JJ , Barentsz JO , Rovers MM . Accuracy of multiparametric MRI for prostate cancer detection: a meta‐analysis. AJR am J Roentgenol. 2014;202(2):343–351. 10.2214/AJR.13.11046 24450675

[12] Trabulsi EJ , Tripathi SK , Gomella L , Solomides C , Wickstrom E , Thakur ML . Development of a voided urine assay for detecting prostate cancer non‐invasively: a pilot study. BJU Int. 2017;119(6):885–895. 10.1111/bju.13775 28075510 PMC5444967

[13] Nerli RB , Ghagane SC , Bidi SR , Thakur ML , Gomella L . Voided urine test to diagnose prostate cancer: preliminary report. Cytojournal. 2021;18:26. 10.25259/Cytojournal_76_2020 34754324 PMC8571200

[14] Wang L , Lu B , He M , Wang Y , Wang Z , Du L . Prostate cancer incidence and mortality: global status and temporal trends in 89 countries from 2000 to 2019. Front Public Health. 2022;10:811044. 10.3389/fpubh.2022.1007528 35252092 PMC8888523

[15] Culp MB , Soerjomataram I , Efstathiou JA , Bray F , Jemal A . Recent global patterns in prostate cancer incidence and mortality rates. Eur Urol. 2020;77:38–52. 10.1016/j.eururo.2019.08.005 31493960

[16] Hessels D , Schalken A . Urinary biomarkers for prostate cancer: a review. Asian J Androl. 2013;15:333–339. 10.1038/aja.2013.6 23524531 PMC3739649

[17] Cristofanilli M , Hayes DF , Budd GT , Ellis MJ , Stopeck A , Reuben JM , et al. Circulating tumor cells: a novel prognostic factor for newly diagnosed metastatic breast cancer. J Clin Oncol. 2005;23(7):1420–1430. 10.1200/JCO.2005.08.140 15735118

[18] Reubi JC , Laderach U , Waser B , Gebbers JO , Robberecht P , Laissue JA . Vasoactive intestinal peptide/pituitary adenylate cyclase‐activating peptide receptor subtypes in human tumors and their tissues of origin. Cancer Res. 2000;60(11):3105–3112. 10850463

[19] Lelievre V , Pineau N , Waschek J , Vaudry H , Arimura A . Pituitary adenylate cyclase‐activating polypeptide. In: Endocrine updates, The biological significance of PACAP and PACAP receptors in human tumors from cell lines to cancer 20 New York: Springer‐Verlag; 2003. p. 361–399.

[20] Ghagane SC , Rangrez S , Nerli RB , Thakur ML , Gomella LG . Use of TP4303 to identify prostate cancer cells in voided urine samples. Can J Urol. 2024;31(3):11893. 38912942

[21] Tripathi S , Trabulsi EJ , Gomella L , Kim S , McCue P , Intenzo C , et al. VPAC1 targeted Cu‐64‐TP‐3805 PET imaging of prostate cancer: preliminary evaluation in man. Urology. 2016;31:29–36. 10.1016/j.urology.2015.10.012 PMC4788593 26519886

[22] Zia H , Hida T , Jakowlew S , Birrer M , Gozes Y , Reubi JC , et al. Breast cancer growth is inhibited by vasoactive intestinal peptide, (VIP) hybrid, a synthetic VIP receptor antagonist. Cancer Res. 1996;56(15):3486–3489. 8758916

[23] Thakur M , Tomar VS , Dale E , Gomella LG , Solomides C , Kolesnikov O , et al. Targeting genomic receptors in voided urine for confirmation of benign prostatic hyperplasia. BJUI Compass. 2024;5(7):675–680. 10.1002/bco2.362 39022663 PMC11250152

[24] Nerli RB , Vinchurkar K , Kalloli M , Rangrez S , Ghagane SC , Thakur ML . Diagnosis of cancer of the cervix by targeting VPAC receptors on exfoliated cervical cells. Braz J Oncol. 2023;19:1–6.

[25] Nerli RB , Ghagane SC , Rangrez S , Chandra S , Thakur ML , Gomella L . Detection of bladder cancer using voided urine sample and by targeting genomic VPAC receptors. Indian J Urol. 2021;37(4):345–349. 10.4103/iju.iju_132_21 34759527 PMC8555562

[26] Nerli R , Kalloli M , Rangrez S , Ghagane SC , Vinchurkar K , Shreya S , et al. Diagnosis of oral cancers by targeting VPAC receptors: preliminary report. Asian Pac J Cancer Prev. 2023;24(5):1711. 10.31557/APJCP.2023.24.5.1711 37247292 PMC10495900

[27] Fütterer JJ , Heijmink SW , Scheenen TW , Veltman J , Huisman HJ , Vos P , et al. Prostate cancer localization with dynamic contrast‐enhanced MR imaging and proton MR spectroscopic imaging. Radiology. 2006;241(2):449–458. 10.1148/radiol.2412051866 16966484

[28] Tanimoto A , Nakashima J , Kohno H , Shinmoto H , Kuribayashi S . Prostate cancer screening: the clinical value of diffusion‐weighted imaging and dynamic MR imaging in combination with T2‐weighted imaging. J Magn Reson Imaging. 2007;25(1):146–152. 10.1002/jmri.20793 17139633

[29] Turkbey B , Pinto PA , Mani H , Bernardo M , Pang Y , McKinney YL , et al. Prostate cancer: value of multiparametric MR imaging at 3 T for detection‐histopathologic correlation. Radiology. 2010;255:89–99. 10.1148/radiol.09090475 20308447 PMC2843833

[30] Abd‐Alazeez M , Ahmed HU , Arya M , Charman SC , Anastasiadis E , Freeman A , et al. The accuracy of multiparametric MRI in men with negative biopsy and elevated PSA level—can it rule out clinically significant prostate cancer? Urol Oncol. 2014;32(1):45e17–45e22. 10.1016/j.urolonc.2013.06.007 PMC4082533 24055430

